# Potent and Selective Carboxylic Acid Inhibitors of Tumor-Associated Carbonic Anhydrases IX and XII

**DOI:** 10.3390/molecules23010017

**Published:** 2017-12-22

**Authors:** Ylenia Cau, Daniela Vullo, Mattia Mori, Elena Dreassi, Claudiu T. Supuran, Maurizio Botta

**Affiliations:** 1 Dipartimento di Biotecnologie, Chimica e Farmacia, Università degli Studi di Siena, via Aldo Moro 2, I-53100 Siena, Italy; cau.ylenia@gmail.com (Y.C.); m.mattia79@gmail.com (M.M.); botta.maurizio@gmail.com (M.B.); 2 Dipartimento di Chimica, Laboratorio di Chimica Bioinorganica, Università degli Studi di Firenze, Polo Scientifico, Via della Lastruccia 3, 50019 Sesto Fiorentino (Firenze), Italy; daniela.vullo@unifi.it (D.V.); claudiu.supuran@unifi.it (C.T.S.); 3 Center for Life Nano Science@Sapienza, Istituto Italiano di Tecnologia, viale Regina Elena 291, I-00161 Roma, Italy; 4 Dipartimento NEUROFARBA, Sezione di Scienze Farmaceutiche, Università degli Studi di Firenze, Via Ugo Schiff 6, 50019 Sesto Fiorentino (Firenze), Italy; 5 Sbarro Institute for Cancer Research and Molecular Medicine, Center for Biotechnology, College of Science and Technology, Temple University, BioLife Science Building, Suite 333, 1900 N 12th Street, Philadelphia, PA 19122, USA

**Keywords:** carbonic anhydrase inhibitors (CAIs), carboxylic acid, tumor-associated isoforms, isoform selectivity, molecular modeling

## Abstract

Selective inhibition of tumor-associated carbonic anhydrase (CA; EC 4.2.1.1) isoforms IX and XII is a crucial prerequisite to develop successful anticancer therapeutics. Herein, we confirmed the efficacy of the 3-nitrobenzoic acid substructure in the design of potent and selective carboxylic acid derivatives as CAs inhibitors. Compound **10** emerged as the most potent inhibitor of the tumor-associated hCA IX and XII (*K*_i_ = 16 and 82.1 nM, respectively) with a significant selectivity with respect to the wide spread hCA II. Other 3-nitrobenzoic acid derivatives showed a peculiar CA inhibition profile with a notable potency towards hCA IX.

## 1. Introduction

Carbonic Anhydrases (CAs; EC 4.2.1.1) are ubiquitous Zn-dependent metalloenzymes that catalyze the interconversion of carbon dioxide (CO_2_) to bicarbonate ion (HCO_3_^−^). To date, 16 isoforms have been characterized in humans and are involved in many physiological and physio-pathological processes, like the regulation of pH and acid–base homeostasis, CO_2_ elimination, bone resorption, electrolyte secretion, gluconeogenesis, ureagenesis, and tumorigenesis [1,2].

Nowadays, there are at least 25 CA inhibitors (CAIs) used in the clinical practice or in clinical development for the treatment of glaucoma [3], epilepsy [4], ulcers [5], infectious diseases [6,7,8], osteoporosis [9], obesity [10], Alzheimer’s disease [6], and cancer [11], which highlight CAs as profitable drug targets for pharmacological intervention. Targeting specific CA isoforms has been indeed exploited in the therapy of different diseases. Human CA II (hCA II), hCA IV, and hCA XII are usually referred as anti-glaucoma drug targets [3,12,13,14], hCA IX and hCA XII are well-known tumor-associated isoforms [15,16,17,18], hCA VA is a promising drug target for the treatment of obesity [19,20,21], while hCA VII is a drug target for the treatment of neuropathic pain and epilepsy [1,22,23,24]. Coupled with ubiquitous expression and with their critical role in cell homeostasis, indiscriminate inhibition of many hCA isoforms at the same time may lead to detrimental side effects. Although the design of CAIs is a consolidated strategy in drug discovery, the development of isoform-selective CAIs still represents one of the major challenges for CA-related medicinal chemistry and drug development [25].

**Figure 1:**
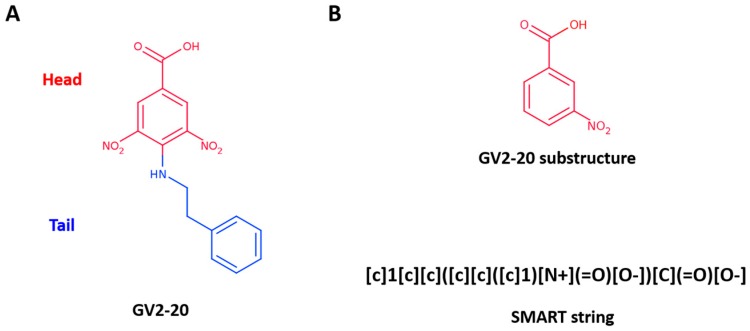
(**A**) GV2-20 chemical structure; (**B**) GV2-20 substructure used to filter MolPort database represented as 2D structure (top) and SMART description (bottom).

Recently, we have established an *in silico* target fishing approach to recycle a false-positive hit (namely GV2-20, Figure 1A) highlighted in a 14-3-3 cell-based screening [26,27,28,29]. Particularly, GV2-20 bears a carboxylic acid group and showed potent inhibition of hCAs with remarkable selectivity for hCA II, hCA VII, hCA IX, and hCA XII. Interestingly, among CAIs with carboxylic acid group, GV2-20 possessed a new scaffold, thus becoming a privileged molecule for further development with potential use as diagnostic and therapeutic tool.

Among established Zn-binding groups such as hydroxamic acid, thiol, phenols, and sulfonamide, carboxylic acid is widely used in substrates and inhibitors of different classes of metalloproteins such as CAs [30,31], matrix metalloproteinases (MMPs) [32,33,34,35], angiotensin converting enzyme (ACE) [36], Zmp1 from Mycobacterium tuberculosis [37], COP9 signalosome subunit 5 (CSN5) [38], and tyrosinase [39] for its ability to coordinate the metal center. Different from well-known chemotypes of CAIs such as for instance sulfamides, sulfonamides, and their derivatives whose interaction with CAs has been extensively characterized by X-ray crystallography [25,40], structural features of potent and selective CAIs based on carboxylic acid is poorly elucidated yet. X-ray crystallography studies performed on hCA II have highlighted two binding sites for carboxylic acid CAIs, located within the catalytic cavity [41], or in a pocket that is commonly referred as “out of the binding site” [42]. Notably, a number of low molecular weight (MW) benzoic acids proved to bind simultaneously to both these sites [43]. Among the catalytic site binders, we can include carboxylic acid derivatives that bind directly the catalytic Zn(II) ion in a tetrahedral geometry, as well as those anchored to the Zn(II)-coordinated water molecule [44,45,46]. An additional class of carboxylic acid precursors that showed inhibition of hCAs are coumarin and thiocumarin derivatives, which are hydrolyzed by the esterase CAs activity, thus partially occluding the entrance of the catalytic cavity, as highlighted by structural studies [47,48]. 

However, despite their potentiality as CAIs, currently available structural data are not sufficient to clarify the requirements of carboxylic acid CAIs for binding to hCAs, which also contributed to explain the lower efforts dedicated to this class compared to widely explored sulfonamides and sulfamides [30].

**Figure 2:**
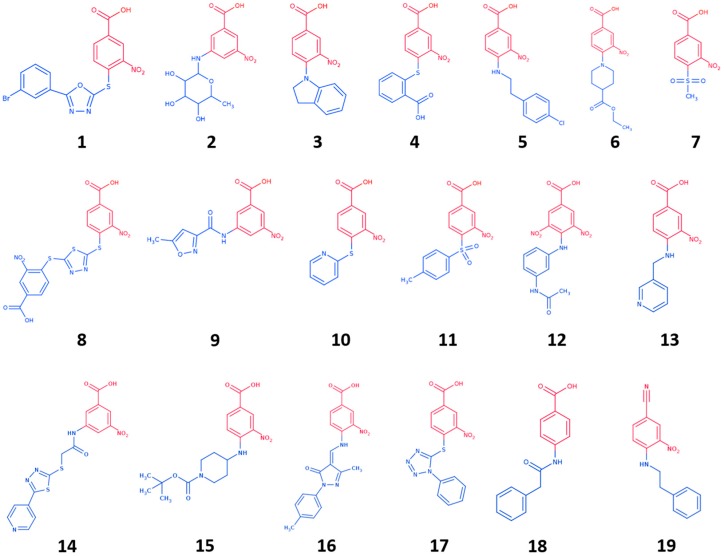
Chemical structure of selected GV2-20 derivatives. Head portions are colored red, tails are colored blue.

To further explore the molecular determinants responsible for potent and selective inhibition of hCAs by carboxylic acids, here we used the tail approach to design a number of derivatives of the previous CAI hit GV2-20. Indeed, this is a versatile tool to design and optimize hit and lead CAIs possibly improving potency and selectivity [49,50]. Our previous results showed that the symmetric 3,5-dinitrobenzoic acid moiety of GV2-20 binds preferentially the catalytic site, thus becoming the head of the molecular scaffold; accordingly, the tail moiety is expected to bind the external and highly variable region of hCAs [51] and enhance selectivity inhibition. Following this strategy, 17 compounds (namely, compounds **1**–**17**, Figure 2) bearing the common 3-nitrobenzoic acid substructure (Figure 1B) and having a significant chemical diversity each other were retrieved by filtration of a commercial database. In addition, two compounds bearing modifications to the head portion of GV2-20 were selected (namely, compounds **18** and **19**, Figure 2) to substantiate the pharmacophoric relevance of the selected 3-nitrobenzoic acid head. Inhibitory properties of **1**–**19** were profiled against a panel of recombinant hCAs. The possible binding mode of the most interesting CAIs in terms of potency and selectivity was investigated by molecular modeling against tumor-associated hCA IX and hCA XII.

Results of this multidisciplinary effort provide additional insights to the possible binding of CAIs bearing carboxylic acid moiety and set the bases for further investigations.

## 2. Results and Discussion

### 2.1. Substructure Search and Ligands Selection

To pursue the tail approach on the previously identified hit GV2-20, we selected the 3-nitrobenzoic acid substructure as head (Figure 1B, top). Indeed, carboxylic acid is a well-known pharmacophore that has been widely exploited in the design of small molecule inhibitors of metalloproteins [52,53].

A fast and efficient way to extract molecules with a specific substructure from chemical libraries is to use SMART- or fingerprint-based filtration. Here we used the SMART string described in Figure 1B (bottom panel) that corresponds to the 3-nireobenzoic acid to extract 1111 commercially available molecules bearing the selected substructure from the MolPort database (consisting of more than 6 million entries at the time of our experiments, April 2016). 

Chemical diversity of filtered compounds was then evaluated, and a subset of nineteen compounds (**1**–**19**, Figure 2) endowed with tails that differ in chemical, shape, and electronic properties from each other was selected for CAs inhibition assays. Tail groups of **1**–**19** include amide, sulfone, sugar, aromatic, aliphatic, heteroaromatic, and heterocyclic moieties. MW of **1**–**19** ranges from 231 to 480. This subset is expected to enhance selectivity and potency of the reference inhibitor GV2-20.

Besides 3-nitrobenzoic acid derivatives and with the aim to evaluate the relevance of the head group in CA inhibition, we also included in our selection compound **18**, which lacks the nitro group in the head, and **19**, where the carboxylic acid group is replaced by a nitrile group.

### 2.2. Carbonic Anhydrase Inhibition

Compounds **1**–**19** were tested for their inhibitory activity against pharmacologically relevant hCA isoforms by means of the stopped-flow CO_2_ hydrase assay [54], using as reference compound acetazolamide (AAZ). hCAs inhibition data are reported in Table 1, together with the values measured previously for GV2-20 [26], which are included in Table 1 to facilitate comparison.

Overall, it is clear from Table 1 that, compared to GV2-20, all tested compounds showed a decreased efficacy against hCA I, hCA II, hCA IV, and hCA VII, and notably improved their potency of inhibition against hCA IX, which contributed to the increased selectivity observed towards this latter isoform.

Further analysis of Table 1 led to draw the following structure–activity relationships (SAR):

(i) All tested compound are poorly active against cytosolic hCA I and hCA II, as *K*_i_ values were significantly higher with respect to the reference compounds AAZ and GV2-20. The drop of inhibitory potency compared to GV2-20 is particularly marked against hCA II, where the most potent inhibitor of the series, namely compound **1**, has a *K*_i_ around 54-fold higher than that of AAZ and around 10-fold higher than that of GV2-20. Sub-micromolar inhibition of hCA I was observed only for compounds **1** and **8**, bearing respectively a 1,3,4-oxadiazole and a 1,3,4-thiadiazole moiety in the tail, while **1** was the only hCA I inhibitor with sub-micromolar *K*_i_. Modifications to the heterocycle such as the tetrazole in **17** proved ineffective for hCA II inhibition. Other tested compounds showed *K*_i_ in the micromolar range, or proved inactive at all against these cytosolic hCA isoforms.
(ii) Similar to the reference inhibitor GV2-20 but different from AAZ, tested compounds proved generally inactive also against hCA IV.
(iii) Compared to GV2-20, most of tested compounds showed a significant improvement of hCA VA inhibition. Notably, the most potent hCA VA inhibitor of the series was **19**, in which the carboxylic acid is replaced by the nitrile function thus opening new venues for the design of hCA VA-specific inhibitors. Other compounds with a low *K*_i_ against this isoform are **10**, **12**, **13**, **15**, and **16** that also show a certain degree of specificity for hCA VA particularly compared to cytosolic hCA I and hCA II, hCA IV, and hCA VII.
(iv) Most of tested compound were poorly active against hCA VII that, in contrast, was efficiently inhibited by GV2-20 and AAZ. As already observed for hCA I and hCA II, compound **1** was the most potent inhibitor of hCA VII, even though the chemically-related compound **8** proved inactive. Compared to GV2-20, compound **1** showed around 37-fold drop of inhibitory potency against hCA VII. Moreover, **5**, **13**, and **19** that are structurally-related to GV2-20 proved inactive. This suggests that subtle modifications to GV2-20 scaffold have a dramatic impact on hCA VII inhibition, and that the 3,5-dinitrobenzoic acid is required to target this hCA isoform.
(v) Tumor-associated isoforms hCA IX and hCA XII were potently inhibited by most of tested compounds. It is worth noting that some compounds showed stronger or at least similar inhibition of hCA IX compared to the reference compounds AAZ and GV2-20, see for example **6**–**8**, **10**, **14**–**17**, whereas none of them was able to overtake AAZ or GV2-20 against hCA XII. The most potent inhibitor of hCA IX was **10**, which also inhibited hCA XII in the low nanomolar range. Unique among other is the behavior of compounds **6** and **7**, which are low nanomolar inhibitors of CA IX with potency comparable to AAZ, although they are not active against hCA XII. Of note, **7** is the lowest MW compound of the test-set, thus becoming the selective hCA IX inhibitor endowed with the highest ligand efficiency identified in this work [55]. Therefore, its structure could be easily expanded by rational design with the aim to optimize physicochemical and pharmacological properties up to the level of confirmed lead or preclinical candidate. Finally, since hCA II is the most physiologically abundant hCA isoform, and is generally referred as the major causes of CAIs side-effects [1], the hCA II/hCA IX and hCA II/hCA XII selectivity indexes are showed in Table 1. Notably, the most potent inhibitors of tumor-associated hCA IX and hCA XII isoforms are also significantly selective with respect to the cytosolic hCA II. While all compounds showed a greater selectivity than the reference inhibitors GV2-20 and AAZ, compound **1** showed the weakest specificity for tumor-associated hCAs, whereas **10** emerged as the most selective one.
(vi) The GV2-20 derivative **18** that is deprived of the nitro group in the head portion is poorly active in most hCA isoforms, with the only exception of hCA IX, for which it showed a *K*_i_ of 72.8 nM. Based on the comparison with the hCAs inhibition profile of GV2-20, we may speculate that at least one nitro group in the head portion is essential for the efficient inhibition of most hCAs. Compound **19** that bears a nitrile instead of carboxylic acid group, shares a similar hCAs inhibition profile as **18** with the exception of a stronger inhibition of hCA VA (*K*_i_ = 92.5 nM).

In summary, SAR analysis highlighted compound **1** as an interesting pan-hCAs inhibitor with a moderate selectivity for tumor-associated hCA IX and hCA XII with respect to cytosolic hCA II and hCA VA, while **10** proved to be an attractive low MW anticancer lead candidate endowed with potent and selective inhibition of hCA IX and hCA XII. The highest efficiency ligand **7** emerged as selective inhibitor of hCA IX, particularly with respect to hCA XII, which paves the way to the design of tool compounds for isoform-specific modulation of hCA IX. The 3-nitrobenzoic acid substructure was confirmed as privileged head for the design of potent and selective carboxylic acid CAIs. 

### 2.3. Molecular Modeling Study

Since the binding mode of carboxylic acid CAIs is highly variable [47], and our crystallization trials were unsuccessful, we performed a molecular modeling study to provide structural support (where possible) to SAR and selectivity profiles of the most interesting hCAIs identified in this work. Based on Table 1, and considering that the parent compound GV2-20 showed antiproliferative effects on chronic myeloid leukemia cells [26], tumor-associated hCA IX and hCA XII isoforms were selected for this task. Compounds **1**, **7**, **10**, **15**, and **19** were docked against the crystallographic structure of hCA IX, while **1**, **7**, and **10** were docked towards hCA XII. Indeed, **1** was an interesting pan-CAs inhibitor with a moderate specificity for hCA IX and hCA XII; **7** was the lowest MW compound showing very potent inhibition of hCA IX with no effects on hCA XII; **10** was the most potent inhibitor of hCA IX showing a good inhibition of hCA XII and the highest selectivity with respect to the cytosolic and widely expressed hCA II; **15** and **19** showed a remarkable potency and selectivity against hCA IX and hCA XII, and were notably active against hCA VA for which no crystal structures are available to date. 

For the sake of clarity, in our study we addressed three different binding modes hypothesis: (i) direct binding of the carboxylic acid group to the catalytic Zn(II) ion; (ii) binding of carboxylic acid group to the Zn(II)-coordinated water molecule; and (iii) binding “out of the binding site”. Results discussed thereafter refer to the most reliable binding poses that were generated according to binding mode hypothesis (ii), namely carboxylic acid CAIs that bind the catalytic Zn(II) ion through a bridging water molecule. Docking poses were then refined by energy minimization in explicit water solvent. 

**Figure 3:**
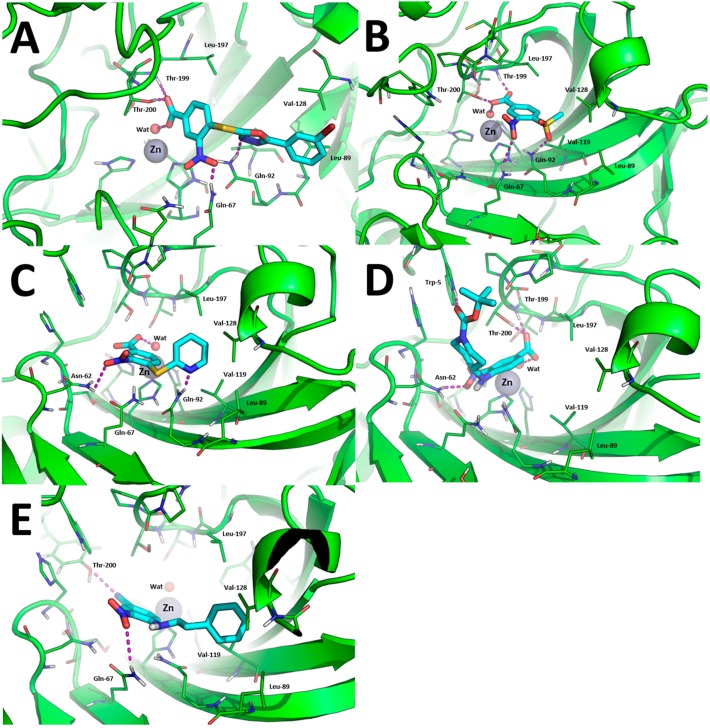
Binding mode of most promising hCA IX inhibitors predicted by molecular docking and energy minimization. (**A**) Compound **1**; (**B**) compound **7**; (**C**) compound **10**; (**D**) compound **15**; and (**E**) compound **19**. Small molecules are showed as cyan sticks, the 3D structure of hCA IX is showed as green cartoon. hCA IX residues involved in binding to compounds are showed as green lines and are labeled. H-bonds are highlighted as magenta dashed lines.

The binding modes toward hCA IX are showed in Figure 3. The carboxylic acid group of **1**, **7**, **10**, and **15** binds the Zn(II)-coordinated water molecule while compound **19**, in which the carboxylic acid is replaced by a nitrile group, does not, which may account for lower inhibition potency of this compound against hCA IX. Besides anchoring the Zn(II)-coordinated water molecule, the carboxylic acid group of compounds **1**, **7**, and **15** performs additional H-bonds with the side chain of Thr-199 and Thr-200 (Figure 3A,B,D). The nitro group of all compounds is H-bonded to the side chain of Gln-67 with the exception of compound **10** and **15** in which it is H-bonded to Asn-62 (Figure 3C,D). The tail groups of compounds **1**, **7** and **10** are H-bonded to Gln-92, while compound **15** establishes an H-bond with the side chain of Trp-5. Moreover, the aromatic ring in the tail of **1**, **10**, and **19** and the methyl group of **8** are docked within a hydrophobic cleft of hCA IX residues formed by Leu-89, Val-119, Val-128, and Leu-197.

**Figure 4:**
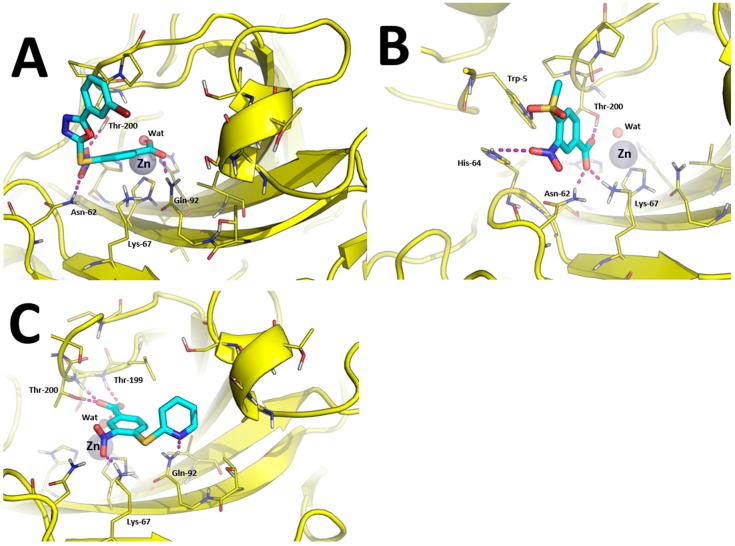
Binding mode of most promising hCA XII inhibitors predicted by molecular docking and energy minimization. (**A**) Compound **1**; (**B**) compound **7**; and (**C**) compound **10**. Small molecules are showed as cyan sticks, the 3D structure of hCA XII is showed as yellow cartoon. hCA XII residues involved in binding to compounds are showed as yellow lines and are labeled. H-bonds are highlighted as magenta dashed lines.

Predicted binding modes towards hCA XII are showed in Figure 4. The carboxylic acid group of compounds **1** and **10** is able to bind the catalytic Zn(II) ion by means of a bridging water molecule (Figure 4A,C). In addition, it is found to bind Gln-92 side chain in the case of **1** and Thr-199 and Thr-200 in the case of **10**. The nitro group of compound **1** is H-bonded with Asn-62 and Thr-200, while the same group of compound **10** binds the side-chain of Lys-67. Moreover, the tail of compound **10** performed an additional H-bond with Gln-92, as already observed in hCA IX (Figure 4C). 

**Figure 5:**
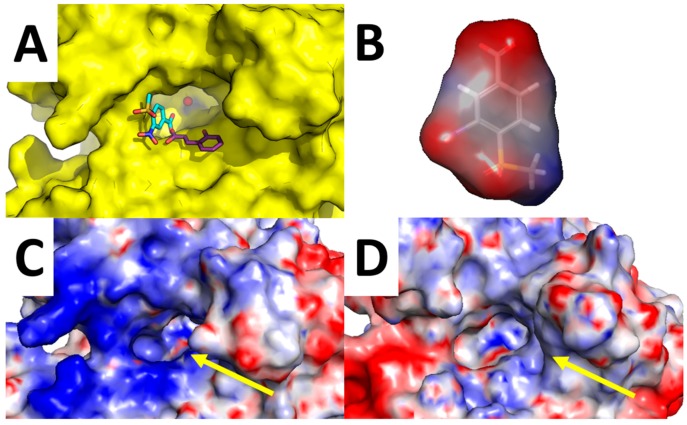
Analysis of compound **7** binding mode. Surrounding of hCA XII Zn(II) binding cavity (highlighted by a yellow arrow) with compound **7** predicted binding-mode and crystallographic hydrolyzed thiocoumarin binding mode (**A**); Surface electrostatic potential of compound **7** (**B**); hCA XII (**C**); and hCA IX (**D**).

Of particular interest is the analysis of the binding-mode of compound **7** because docking simulations were unable to place this molecule in the proximity of the catalytic Zn(II) ion, nor the Zn(II)-coordinated water molecule, as observed instead for other compounds (Figure 3A–D and Figure 4A,B). The carboxylic acid group of **7** is H-bonded to the side chain of Asn-62, Lys-67, and Thr-200, while the nitro group performed an additional H-bond with His-64. Concerning the tail of the molecule, the sulfone group of **7** is H-bonded to Trp-5. As shown in Figure 5A, **7** is unable to occlude the catalytic site of hCA XII, which is still accessible from the solvent area. Accordingly, we speculate that hCA XII enzymatic reactions may still occur with compound **7** bound to the surface of hCA XII**,** thus explaining the lack of inhibition by **7** observed up to high concentrations (see Table 1). Moreover, the predicted binding mode of **7** is in agreement with the binding mode already described for thiocoumarins [47]. As shown in Figure 5A, the carboxylic acid group of the hydrolyzed thiocoumarin partially overlaps with that of compound **7** and does not reach the catalytic Zn(II) coordination system, which strongly corroborates the binding mode predicted by molecular modeling. 

To further strengthen this hypothesis, we calculated the electrostatic surface potential of compound **7**, hCA XII, and hCA IX (Figure 5B–D). As highlighted in Figure 5C,D, the surface of hCA XII near the entrance of the catalytic cavity is positively charged, especially because of basic residues Lys-4, Lys-19, His-64, Lys-170, and His-234, while the corresponding area in hCA IX is negatively charged. Since **7** was docked within the basic surface of hCA XII, it is likely that it is trapped in this region by electrostatic interactions, thus becoming unable to inhibit the catalytic function of the enzyme. 

## 3. Experimental Protocols

### 3.1. Selection of GV2-20 Derivatives and Molecular Modeling

Smiles representations of 6504839 commercially available compounds were downloaded from the MolPort database (interrogated in April 2016). Molecules were subsequently filtered using the Filter program from OpenEye (version 2.5.1.4) [56,57] by using the following SMART string [c]1[c][c]([c][c]([c]1)[N+](=O)[O−])[C](=O)[O–] (Figure 1B, bottom panel). After filtration, a library of 1111 compounds was obtained.

Compounds selected for CAs inhibition assays were purchased from MolPort and tested as received.

Molecular docking of most interesting compounds was performed with GOLD program (version 5.2.1) [58]. The crystallographic structure of hCA IX isoform (PDB ID: 3IAI) at 2.2 Å resolution [59] and hCA XII (PDB ID: 1JCZ) at 1.55 Å resolution [60] were used as rigid receptors in the docking program. To account the three different binding hypothesis described in Section 2.3, the binding site was centered on different positions: for hypothesis (i) and (ii), it was centered on the catalytic Zinc(II) ion, while in the hypothesis (iii), it was centered on His-64, ND1 atom. The radius of all binding sites was set to 14 Å. For each ligand, 30 runs of the Genetic Algorithm (GA) were performed. The CHEMPLP scoring function was used and the GA search efficiency was increased up to 200%. Moreover, in the binding mode hypothesis (ii) the water coordinated to the catalytic Zn(II) ion was added in the hCA IX/hCA XII structure by aligning with the crystallographic structure of hCA II isoform (PDB ID: 4QY3) at 1.5 Å resolution, which bears a crystallographic Zn(II)-coordinated water molecule bound to carboxylate ion. During docking with GOLD program, the toggle state of the Zn(II)-coordinated water molecule was set to “on”.

Ligands protonation state was assigned by fixpka application from QUACPAC (version 1.6.3.1, OpenEye software) [61]. Finally, ligand energy minimization before docking was performed with SZYBKI (version 1.8.0.1, OpenEye software).

The best docking-based binding pose of each compound was subsequently relaxed in explicit water solvent with AMBER 16 [62]. The AMBER force field ff14SB [63] was used for standard amino acids, while Zn(II) ion was modeled using the cationic dummy atom model [64]. Gaff2 force field was used for small molecules parameters [65]. Each system was solvated by a 10 Å cuboid box of explicit TIP3P-type water molecules. To neutralize the whole charge, the required number of Na+ counterions was then added. Water molecules were first minimized (1000 steps with the steepest descendent algorithm (SD) and additional 2000 steps with the conjugate gradient algorithm (CG)), while the protein was kept frozen. Then, the whole system was minimized by means of 1500 steps of SD and 13,500 steps CG.

The electrostatic surface potential of hCA IX and hCA XII was calculated with APBS 1.4.1 with the nonlinear Poisson−Boltzmann equation using the default parameters [66]. 

Figures describing ligands binding modes and electrostatic surfaces of compound **7** and hCAs were generated with PyMol [67].

The electrostatic surface potential for compound **7** was calculated using the Schrödinger’s Maestro Suite for molecular modelling by solving the Poisson−Boltzmann equation and using the partial charges of the molecules **7** [68].

### 3.2. CA Inhibition

An applied photophysics stopped-flow instrument has been used for assaying the CA catalysed CO_2_ hydration activity [54]. Phenol red (at a concentration of 0.2 mM) has been used as indicator, working at the absorbance maximum of 557 nm, with 20 mM Hepes (pH 7.5) as buffer, and 20 mM Na_2_SO_4_ (for maintaining constant the ionic strength), following the initial rates of the CA-catalysed CO_2_ hydration reaction for a period of 10–100 s. The CO_2_ concentrations ranged from 1.7 to 17 mM for the determination of the kinetic parameters and inhibition constants. For each inhibitor, at least six traces of the initial 5–10% of the reaction have been used for determining the initial velocity. The uncatalysed rates were determined in the same manner and subtracted from the total observed rates. Stock solutions of inhibitor (0.1 mM) were prepared in distilled–deionized water, and dilutions up to 0.01 nM were done thereafter with the assay buffer. Inhibitor and enzyme solutions were preincubated together for 15 min at room temperature prior to assay, in order to allow for the formation of the enzyme–inhibitor complex. The inhibition constants were obtained by non-linear least-squares methods using PRISM 3 and the Cheng–Prusoff equation, as reported earlier [69,70,71], and represented the mean from at least three different determinations. All CA isoforms were recombinant ones obtained *in-house* as reported earlier [70,71,72,73].

## 4. Conclusions

Nowadays, the development of isoform-selective CAIs has become a major focus in the discovery of innovative therapies for a wide range of human diseases.

Starting from the pan-hCAs inhibitor GV2-20, in this study we performed a tail approach in order to improve potency and selectivity of inhibition among hCAs. Notably, almost all tested compounds showed potent inhibition of hCA IX and increased selectivity with respect to other isoforms such as the widely expressed cytosolic hCA I and hCA II. Of note, compound **10** emerged as the most promising inhibitor of the tumor-associated isoforms hCA IX and hCA XII with an impressive hCA II/hCA IX and hCA II/hCA XII selectivity index, while compound **7** was a low nanomolar and highly selective inhibitor of hCA IX with the highest ligand efficiency. Finally, the 3-nitrobenzoic acid was confirmed as a good scaffold for the design of CAs inhibitors. 

## Figures and Tables

**Table 1:** Inhibition data of hCA I, hCA II, hCA IV, hCA VA, hCA VII, hCA IX, hCA XII with compounds **1**–**19**, GV2-20, and the standard sulfonamide inhibitor acetazolamide (AAZ) by a stopped flow CO_2_ hydrase assay.

Cmpd	*K*_i_ (nM) ^a^							Selectivity Index ^b^	
	hCA I	hCA II	hCA IV	hCA VA	hCA VII	hCA IX	hCA XII	hCA II/hCA IX	hCA II/hCA XII
**1**	700	655	5090	826	325	55.1	27.3	11.88	23.99
**2**	5645	7240	>50,000	1352	>50,000	106	>50,000	68.3	-
**3**	>50,000	7300	>50,000	1314	840	209	>50,000	34.9	-
**4**	6020	2630	>50,000	>50,000	>50,000	172	>50,000	15.3	-
**5**	8150	>50,000	>50,000	1163	>50,000	153	863	>326.8	>57.9
**6**	8210	8050	>50,000	>50,000	>50,000	30.6	>50,000	263.1	-
**7**	>50,000	7310	>50,000	1456	>50,000	27.6	>50,000	264.9	-
**8**	6450	801	8800	>50,000	>50,000	26.3	912	30.4	0.9
**9**	>50,000	8500	8270	>50,000	762	165	753	51.5	11.3
**10**	6650	>50,000	>50,000	142	665	16.0	82.1	>3125	>609.01
**11**	>50,000	>50,000	>50,000	356	>50,000	58.3	778	>857.6	>64.3
**12**	>50,000	>50,000	>50,000	144	>50,000	82.0	91.9	>609.0	>544.1
**13**	>50,000	>50,000	>50,000	117	>50,000	106	633	>471.7	>79.0
**14**	>50,000	>50,000	>50,000	449	>50,000	23.0	482	>2174.0	>103.7
**15**	>50,000	>50,000	9200	110	>50,000	24.7	724	>2024.3	>69.1
**16**	>50,000	>50,000	6030	154	1733	30.1	352	>1661.1	>142.0
**17**	>50,000	>50,000	3560	739	>50,000	23.4	441	>2136.8	>113.4
**18**	2730	7630	>50,000	262	>50,000	72.8	629	104.8	12.1
**19**	>50,000	>50,000	>50,000	92.5	>50,000	79.1	648	>632.1	77.2
GV2-20	352	67.3	7660	895	8.7	42.3	9.6	1.59	7.01
AAZ	250	12.1	74.3	63.5	2.6	25.0	5.7	0.48	2.12

^a^ Errors in the range of ± 5–10% of the reported values, from 3 different assays (data not shown); ^b^ Selectivity index was not calculated for compounds showing *K*_i_ > 50,000 nM for inhibition of hCA XII.
